# A Thesis on Typhoid Fever, Presented to the Faculty of the Oglethorpe Medical College, at Savannah, Ga., Session 1855 and 1856

**Published:** 1856-08

**Authors:** Jonathan J. Jones

**Affiliations:** Knoxville, Ga.


### ARTICLE II.

*A Thesis on Typhoid Fever, presented to the Faculty of the Oglethorpe Medical College, at Savannah, Georgia. Session* 1855 *and* 1856. By JONATHAN J. JONES, of Knoxville, Georgia. Published by the Faculty.

### *Typhoid Fever.*

GENTLEMEN:—I have not selected Typhoid Fever as the subject of my dissertation, thinking I was capable of throwing any additional light on the subject, obscure as it is, but from the fact, that I have resided in a region of country during the last five years, in which it has extensively prevailed, and that I have given it as much, probably more, attention than any other disease with which I have had to contend. It has scourged Middle Georgia for the last ten years: many men who were bound to earth by all the endearing social ties of life, whose public and private services to the country were inestimable, have fallen victims to this disease: many good and kind women, who gave the examples of virtue, benevolence and Christianity to the world in an eminent manner, and who fulfilled the noble mission of woman, as mother, or as wife, to cheer man's gloomy pathway through all the vicissitudes of life, to lighten his burdens, and render home delightful with their smiles, have ceased to be, and are numbered with the dead —the work of this fell destroyer, Typhoid Fever. It has spared no sex or condition, and has shown its malignancy, alike in the palace and the hovel. When and where it makes its appearance, it brings consternation and alarm; for well has experience taught the people, that death follows in its footsteps. Often has it fallen to my lot to witness its insidious attacks, and see its victims go step by step, slowly, but surely, to the grave, without being able to do more than palliate their sufferings. I know of no disease in the whole catalogue of human ills, that is better calculated to humble the scientific and conscientious physician, and cause him to feel more the inadequacy of his means to repel the monster death. As I retrospect the past, I can call to mind many interesting young men who possessed all the requisite qualifications for greatness—who were the pride of fond and indulgent parents, causing their hearts to leap for joy, as they surveyed the future, and thought they saw their children's renown and usefulness. But, alas! how soon have all those fond anticipations been blighted by the appearance of Typhoid Fever, which dimmed the bright eyes—made pallid the intelligent countenances of their loved ones, and hushed their voices in the stillness of death forever. My heart almost bleeds as I reflect on those scenes—scenes in which I had to stand a "quiet looker-on," without the means to prevent the approach of death, although I had at my command the whole materia medica. But this is looking upon the dark side of the "picture." When Typhoid Fever prevails, and many persons are attacked, some *will* die; but if proper medical attention is given them, many that would otherwise die, may be saved.

Typhoid Fever is a self-limiting disease. No medicine—no course of treatment that I have ever seen used, or brought to bear upon it, has had any effect towards shortening the disease. It is emphatically a slow fever, taking several days, and sometimes even weeks, for its development. I have seen cases in which it was preceded by its premonitory symptoms, two, and sometimes three weeks before the individual attacked, would leave off pursuing his usual avocation, and when he left it off, said that he was induced to do so only from extreme weakness.

I have seen but few cases of Typhoid Fever in which the patients complained of much pain; and I believe, as a general rule, that they experience but very little after the development of the fever. It is, however, preceded, generally, by headache, and pains in the back and limbs, and a large number of other unmistakable *nervous symptoms* mark its incipiency. Such as prostration of strength, stupor, restlessness, &c.

I propose, gentlemen, in this dissertation, to speak of Typhoid Fever as I have seen it, and not as it is spoken of in the books; therefore, when I make an assertion I desire that you understand me as saying, the fact asserted is so only according to my experience.

The headache that precedes Typhoid Fever is of a peculiar character. It is a dull, constant pain, accompanied with ringing in the ears, and is almost always confined to the posterior portion of the brain. I do not recollect that I ever heard a patient complain of pain in the frontal region, and believe that it seldom occurs in that portion of the brain. I noted the symptoms of over thirty cases that I treated during the fall of 1855, and in every case the pain was in the posterior and basilar region of the brain; and in thirteen of that number, it extended down the neck, and was severest in the medulla-oblongata. The patient is dull and listless from the beginning of the disease, and manifests an almost utter indifference for every thing and every body. You will probably hear him complain of debility on your first visit to him. It is one of the first symptoms complained of—in fact, it is a premonitory symptom, showing the early implication of the nervous system in Typhoid Fever. The disease is generally ushered in by a chill, followed by the usual phenomena of fever. This may occur, as I before remarked, several days, or even weeks, after the observance of the nervous symptoms mentioned, but usually in four or five days. The fever that follows the chill is generally of the continued type, and all the authors whose works I have had an opportunity of examining, say, that it is always a continued fever, but I have seen several well marked cases of Typhoid Fever that were remittent, and some that were even intermittent in their characters; but the intermissions in the intermittent types, were not so complete as they are in the ordinary forms of intermittent fever.

The pulse at the commencement of the fever is full and hard, and continues so during the first few days, (though this is not always the case, sometimes it is weak, small and rapid from the first,) it then grows gradually weaker, smaller and more rapid, and greatly accelerated upon the slightest exertion made by the patient. Some authors say the pulse is usually under a hundred beats in the minute, but I have not found it so. I have generally found the pulse of an adult male, at the expiration of the first week of the fever, at from 110 to 120, and that of an adult female, at from 120 to 130. The tongue is covered at first with a dingy white coat, which in two or three days becomes brown, and during the second week black. The teeth are also covered with dark sordes about the same time. About the middle of the first week there is a disposition to diarrhœa, and unless the physician is particular in the administration of purgatives, his patient will be exhausted by large watery evacuations, which frequently set in without any warning, and in a few hours reduce the patient to the brink of the grave. I have several times seen the above effects produced by a single blue pill; the purgative action of which, the physician did not think it necessary to guard with opium. A day or two before this tendency to diarrhœa occurs, if the right iliac region be examined, it will be found tender to the touch, and distended with gas, which produces a gurgling sound when pressed upon. This symptom is observable through the whole course of the disease, until convalescence, or until the bowels become generally tympanitic, which usually occurs before the patient dies. Authors who have written on this subject say, that about the seventh or eighth day of the fever, symptoms of pneumonia or bronchitis make their appearance; but I have seen very few cases in which pneumonia or bronchitis appeared after the commencement of the fever, if there was no irritation of the lungs, or bronchial lining *previous* to the commencement. These symptoms, then, are not constant attendants on the fever, but are only occasionally to be met with.

During the second week, delirium in most instances supervenes. It generally comes on at first, during the night only, and appears immediately after the patient has been aroused by a disturbed sleep. In this case of typhomania he seems inattentive to everything around him, and mutters like one talking in dreams. The delirium is not usually violent, but on the other hand, the patient is remarkably quiet—lying in a half comatose state, from which, however, he can generally be aroused, and when aroused is rational, but when left alone, soon relaxes into his former condition. While the patient is in this state, the nervous system is very obtuse; he is dull of hearing; the sense of smell is very imperfect; neither can he taste anything, but will take the most nauseous substances without complaining of them; also the sense of feeling is very much blunted; flies may crawl over his face and hands without exciting any attention to them. At this period of the disease, there is a strong tendency to diarrhœa, and frequently the very best treatment will not prevent its occurrence. The evacuations are either large and watery, of a yellowish color, mixed with shreds of mucous membrane, or small, and mixed with blood, resembling the dejections in dysentery. It is now of the utmost importance that the bowels be controlled, and *that* physician, whose means will best effect this object, will cure the greatest number of cases of Typhoid Fever. The eruption now makes its appearance on the breast, bowels and back of the patient. The eruption consists of spots, about a line in diameter, and of a rose color, which fade upon pressure. Dr. Watson, in his work on the practice of medicine, denies this last assertion; but I have frequently seen them disappear by merely rubbing the finger over them. They would, however, re-appear again immediately on the removal of the finger. These spots are certainly characteristic of Typhoid Fever. I do not recollect to have seen a case in a white person in which they were absent; though a large majority of the cases I have attended were negroes, and owing to the color of the skin, I was unable to detect the eruption on them; but as it was to be seen on all, or nearly all of the whites, on whom I have attended, I must think it was present also, in the negroes' cases, as a large majority of the other symptoms were identical.

Another eruption is mentioned by writers, called *sudamiina.* They are, says Dr. Watson, small, hemispherical, transparent elevations of the cuticle, containing a clear watery fluid. The vesicles are about a quarter of a line in diameter; they have no red basis, and are so perfectly pellucid that when you look upon them in a direction perpendicular to the skin on which they stand, they may readily elude observation. Viewed sideways they present bright surfaces, and look like so many drops of water, and you may feel with your hand that they *roughen* that part affected with them. These sudamina are mostly to be met with on the thorax, along the sides of the neck, and about the axilla. By degrees, the limpid fluid disappears, and they shrink up, the cuticle becomes wrinkled and dries into a white powder. I have met with this eruption but twice, although I have seen several hundred cases of Typhoid Fever. So I must think that it is an exceedingly rare symptom. It is said to appear between the tenth and fourteenth day of the fever, but in one of the cases in which I saw it, it appeared on the twenty-fifth day, and in the other on the twenty-second. Its rare occurrence makes it almost worthless, as a diagnostic symptom, and I cannot say that it is of any value to the physician as a prognostic sign. One of the cases in which I observed it, died, the other recovered.

I have mentioned, I believe, all the symptoms that are usually met with in the first and second weeks of the fever. I will now proceed to the third: This is decidedly the critical period of the disease. I have known but few cases to die during the first or second week; but during the third, at least three-fourths of the deaths that I have witnessed occurred. Although the third week is the critical period in Typhoid Fever, yet all cases do not come to a crisis during that week, but many are protracted to the fourth, others to the fifth, and some to even as late as the sixth and seventh weeks. If a patient dies during the first or second week of the disease, you will generally find that death was produced by perforation of the intestines, which may be recognized by sudden and intense pain in the abdomen, vomiting, syncope, costiveness, and coldness of the extremities, &c.

You will observe at the commencement of the third week, if the case is to terminate unfavorably, a general aggravation of the symptoms, the eyes are injected, the tongue is dry and black, or peels off, leaving the surface intensely red and shining; the pulse is feeble and frequent, sometimes running up to 140 or 150 beats in the minute. There is also low muttering delirium, with *subsultus tendinum* and *floccitatio.* As the debility increases, the patient slips down towards the foot of the bed, hemorrhages take place from the nose or bowels, and also involuntary evacuations from the bowels. The skin is covered with profuse perspiration, and the patient has retention of urine. These are generally the precursors of death, though they are by no means infallible signs; for I have seen patients recover after they had all been observed—"when no one dared to hope." It is a truthful saying, so far as Typhoid Fever is concerned, "that there is hope as long as there is life." If the case is to terminate favorably, you will observe a mitigation of the symptoms, the tongue cleans, and becomes moist, the patient is less dull, the countenance being expressive of more intelligence, his pulse is not so frequent, and the evacuations from the bowels are billious and more consistent. The commencement of the stage of improvement is marked by no critical discharge, but is gradual and sometimes almost imperceptible for several days.

It is during the third week of enteric fever, that bed-sores are usually observed. They are to be found principally on the back and hips of the patient, and are produced, it is said, by lying too much on the back, which the patient is compelled to do from extreme debility. They often cause convalescence to be tedious, and it is sometimes the case that the patient becomes exhausted and dies alone from their debilitating effects; but as a general rule, recovery takes place after their appearance. I do not subscribe to the doctrine that they are produced by lying on the back, for I have seen patients in other diseases lie for a great length of time on the back, and if proper attention was given them, they did not have bed-sores; and, further, if they were produced by the position of the patient in bed, they would always appear on the most prominent parts of the back and hips, as is the case when they *do occur* in other diseases; but I have seen them frequently on parts of the body that could not possibly have been pressed or irritated by the bedding, while the skin on the prominences of the hips and spine was uninjured. They are always preceded by deficient nervous action which I believe to be the prime cause of them, influenced to some extent, probably, by the state of the blood. The patient does not complain of them, therefore the physician should notice for them daily, that by timely attention excessive sloughing may be prevented.

But few writers make any distinction between Typhoid and Typhus Fevers, only that one is a more malignant grade than the other, of continued fever. Dr. Watson says, "there is no line of genuine distinction between continued fevers that can be relied on; they run insensibly into each other, even the most dissimilar of them, and are traceable often to the same contagion." Others of equal eminence say the same. But, notwithstanding the array of evidence they advance to prove their position, I must still believe that Typhoid Fever, although it may be a continued fever, is a disease *sui-generis*—that it is a disease produced by a specific cause; or, in other words, the cause that will produce Typhoid Fever will not produce any other fever. I have never seen a billious fever, or the synochal fever of Cullen, run into a typhoid or enteric fever. It is true, however, that I have seen billious symptoms at the commencement of Typhoid Fever; and I have also seen it commence with symptoms of active inflammation, but a close observer could distinguish characteristic symptoms enough to rightly diagnose the disease.

The only affection to which it bears any resemblance is typhus, and there is such a marked difference in the symptoms of the diseases, that there is, generally, but little difficulty in distinguishing them. Dr. Wilks says, in a report of the cases that occurred in Guy's Hospital in 1854, "I say then, as regards the Typhus and Typhoid genera, that in the majority of instances the two are plainly distinguishable; and that in a few cases in which some doubt may arise, on account of obscurity of the rash or other symptom, a light will subsequently be thrown upon them by the completion of the history, and it will then be clearly seen to which type they belong, and if there be yet, one or two cases in any given period, concerning which an obscurity hangs throughout the whole duration of the illness, they will probably be fewer in number than those instances in which an equal doubt may exist as to the presence of fever or a local inflammatory disease of the head or chest." I am convinced that Typhoid and Typhus Fevers are separate and distinct diseases, by the following circumstances—Typhoid Fever never reaches a crisis under three weeks, or twenty-one days, and is remarkably insidious in its attacks; the patient not becoming very ill before the tenth day. I have watched the disease closely, and almost constantly, during the last five years, and have not seen a case convalesce before the end of the third week. It is true, that I have seen many cases in which considerable improvement had taken place before that time, yet as there existed more or less fever up to that period, it could not be said of the patient that he was fairly convalescent. While on the other hand, on the third or fourth day in typhus, the patient is exceedingly ill; his muscular powers are gone, and his nervous system prostrated. You will, in all probability, find him delirious, and his skin covered with rash, even at that early period of the disease. It comes to a crisis on the thirteenth or fourteenth day. There are, doubtless, exceptions to this rule, but not more than to any other relating to the termination of diseases. The rash in Typhoid Fever makes its appearance between the tenth and fourteenth days of the disease, while in typhus it appears between the fourth and seventh days. There is, also, great difference in the appearance of the two rashes—that of Typhoid Fever being small, leuticular, rose colored spots, about a line or line and a half in diameter, and which fade on pressure. They are slightly raised above the common surface of the skin, and are confined to the bowels, breast, and back of the patient. That of typhus consists of larger spots, of a redish-brown or dark color, confined to no particular locality, and which do not fade upon pressure, being petechial in their character. It is certainly sometimes difficult to distinguish the two rashes, but they are rare and exceptional cases; for in a large majority of cases, the two rashes are well marked, and cannot be confounded. Again, in Typhoid Fever, there is tenderness of the abdomen, with more or less tympanitis, particularly in the right illiac region. These are constant symptoms in this disease, but they are only occasionally observed in typhus. Also, in the former disease, there is diarrhœa, or at least, a strong tendency to it, evidenced by the great susceptibility of the patient's bowels to the action of purgatives. This symptom is present at the beginning of the disease, and is observed throughout its whole course; while in the latter, constipation generally exists, and when an action on the bowels is produced, it is more consistent, darker, and more offensive. And hemorrhage, which frequently takes place in the advanced stages of enteric fever, seldom takes place in typhus. Also the epistaxis that so often occurs at the commencement of Typhoid Fever, does not occur in typhus, and in the latter disease there is more stupor; the surface is darker, or more dusky; the eyes are more turbid, and the prostration is much greater. The anatomical characters of the two diseases are also different. The ulceration of the glands of Peyer, and of the mesenteric glands, so universally found in Typhoid Fever, is never found to exist in typhus, or if found, so seldom as to lead to the suspicion of a complication of the two diseases, when it does occur. The heart, liver, spleen and kidneys are not found enlarged, and softened in typhus, but they are frequently found so conditioned in enteric fever. The former disease often attacks old persons, but the latter never does—the young are always its victims. Persons over thirty years of age appear to be almost exempt from attacks of Typhoid Fever, while they are very subject to attacks of typhus. Typhoid Fever is never epidemic in this country, but is always endemic, while the reverse is the fact with typhus. And, lastly, enteric fever never terminates by any critical discharge, either by sweat, urine, or stool, but one or the other of those discharges mark the approach of convalescence, in almost every case of typhus. Now, with this array of evidence before him, who can say that these two diseases are but modifications of continued fever? Such an one must have the bump of firmness largely developed on his cranium, or be the blind devotee of some cherished theory.

It is the opinion of many physicians of my acquaintance, as well as many writers whom I have consulted on this subject, that Typhoid Fever is contagious. Such an impression having gone forth has been productive of much injury to those afflicted with the disease, by preventing those who were well from giving the sick that attention which was necessary for their comfort and restoration to health. I am sure, however, that it is not contagious, and am induced to think so, from the following facts: I have attended over two hundred cases of the Fever, and have visited my patients under every variety of circumstances. I have sat by their bed-side in the day time, and in the night, for hours, and I have not been attacked by the disease. I have seen others do the same thing, and they have likewise escaped. I have seen the sick removed into families in which the disease did not exist, and have never known it spread under those most favorable circumstances. Does the doctrine of contagion account for this? I think not. Single cases of Typhoid Fever arise at the same time in places remote from each other, and among persons with whom there has been no intercourse whatever. It also often attacks many persons at the same time where it has not previously existed. So, "with the lights before me," I am compelled to believe, that Typhoid Fever is not contagious. It is generally acknowledged by the profession, that we know nothing of the cause of Typhoid Fever, yet there exists a variety of opinions on the subject. Some believe that it is generated by a poison exhaled from human bodies, where many persons are crowded together in jails, houses and ships, that are not sufficiently ventilated. And some, that it is produced from marsh effluvia. Others, that it is produced by a vitiated state of the atmosphere; from the putrefaction of dead animal and vegetable matter; and others still, that it originates in an animal poison, and is contagious.

As an objection to the first proposition, I will quote from Watson's Practice, page 974—"The natives of the arctic regions, who, in order to shelter themselves against the extreme cold of their climate, live, during the greater part of the year, in close subterraneous dwellings, from which the fresh air is studiously excluded, and in which the atmosphere becomes so offensively foul as to be scarcely supportable by a stranger; yet, continued fever is not known among them." It may be said that the coldness of the climate may prevent the propagation of the disease, but Typhoid Fever is a disease that often prevails in cold climates—we have it with us in the winter, and it is equally as malignant as when it occurs in the summer or fall. But the same author gives another example that puts to rest this argument: "A similar exemption from that disease, (continued fever,) is observed within the tropics in the African slave ships where the poor wretches are crowded together below the deck, as close as they can possibly lie, in a sultry climate, barred down with iron to prevent insurrection." Many examples of a similar character might be adduced, as the medical profession have given the subject considerable attention, but I do not choose to weary you with their narration, deeming the above sufficient to convince the most incredulous, if taken in connexion with their own observation. The belief that enteric fever originates from marsh effluvia, is so palpably absurd, according to my understanding of its nature, that it does not admit of an argument. I believe, however, that the two causes, marsh effluvia, and the cause of Typhoid Fever, may both act at the same time, on the same individual, producing a Typhoid Fever of a remittent, or intermittent character. I have often seen Typhoid Fevers of that description, and can account for them on no other principle. I also think it improbable that a vitiated state of the air, from the putrefaction of dead animal and vegetable substances, can give rise to enteric fever. If such was the case, medical students who frequently spend much of their time in the foul air of a dissecting room, would certainly contract the disease, but we find them as free from it as any other class of persons in the country. And, also, persons whose callings in life, places them in situations where they are constantly surrounded by the foul air arising from the putrfeaction of such substances, would be constantly dying of the fever. But such is not the case; then, that cannot be the cause of it. From the belief, that it originates in an animal poison, and is contagious, I must also dissent—my reasons have been previously given.

The circumstances mentioned may, and doubtless do, act frequently as predisposing causes. They are capable of producing debilitating effects, and when the individual has imbibed the true exciting cause, which may not be able to bring the system into subjection to it, they may, by the influence they are capable of exerting, enable this true exciting cause to act, and by that indirect means be the cause of Typhoid Fever, but never in any other way. Dr. Watson says, "however paradoxical the assertion may seem, a predisposing cause may even be applied, and operate, after the exposure to the exciting cause, and so render the latter effective when it might not otherwise have been so."

What, then, can be the exciting cause of Typhoid Fever? It is, doubtless, a specific poison; but how that poison is generated, must, I suppose, ever remain a mystery, as we possess no chemical test by which we are capable of detecting it. As many others have given their views on the subject, and as it is the privilege of every one to think for themselves, and speak their convictions, if they operate to the injury of no one in this golden republic, I, with diffidence, will give mine. I believe that Typhoid Fever is produced by a *poisonous gas, generated by chemical action in such substances as usually accumulate about human residences.* I am induced to adopt this opinion by the following facts: I have frequently seen Typhoid Fever prevail at some particular residence for months at a time, or until nearly every member of the family who resided there were attacked, while the inhabitants of every other residence in the surrounding country were free from the disease. I have frequently seen the fever subside after the removal of such accumulations, and the scattering of chlorinated lime, or other disinfecting agent about the houses and yards. And I have invariably seen it subside when the inhabitants were removed from the infected houses. A family residing in this county had suffered from Typhoid Fever near three months, during which time about thirty of its members had had the disease: new cases were daily occuring, and as there were many yet to have the disease, and every effort had been made, but without the least success, to stop it, a removal was recommended. It was immediately carried into effect. Small tents were pitched about a mile off in the open wood, and notwithstanding those who had not taken the disease were with the sick equally as much after the removal as before, not another case occurred, and the disease in those afflicted soon assumed a mild form, and every one recovered. The family continued to occupy the tents until the houses were renovated, the yards cleansed, and lime freely scattered about the premises, after which they were removed back to the residence, and have not suffered from the disease since, although several years have elapsed. I could mention many circumstances of like character, but the fact is certainly well established, that Typhoid Fever frequently attaches itself to certain isolated places, and that they can be frequently disinfected by certain means, which is all that the foregoing circumstances go to prove. I will tax your patience no farther, feeling, however, confident that I have, at least, good circumstantial proof of the correctness of my position as to the cause of Typhoid Fever. Now, what can the character of this *gas* be, that I have said was the cause of enteric fever?

Our minds are at once directed to carbonic acid. But carbonic acid must be present in considerable quantities where many persons are crowded together in ill-ventilated apartments, and it is also generated by the putrefaction of dead animal and vegetable matter; neither of which causes, I have contended, will produce the disease. I am free to admit that I am unable to conceive of its nature; but taking all the circumstances in connexion, I must think my conclusion is correct—that it is produced by a poisonous gas or effluvia arising from the decomposition of such substances as accumulate about human residences. When we take into consideration the vast quantity, as well as *variety* of such substances, it is not unreasonable to suppose that their decomposition will give rise to poisonous and deleterious gases that are productive of disease, and certain states of the atmosphere may set in action the process which gives rise to this peculiar emanation, which is the cause of Typhoid Fever. But those states of the air most favorable for its production I am unable to tell. The disease prevails in middle Georgia, late in the fall and beginning of winter.

I will call your attention in the next place, to the anatomical characters of this disease; and, as I have opinions relative to them that you may regard as peculiar, I desire your attention particularly to this part of my subject. Post-mortem examinations have revealed the fact, that almost every organ in the human system may be diseased in enteric fever. The liver, heart, spleen and kidneys are frequently found enlarged and softened; in fact, can scarcely be handled without rupturing them. The lungs are also usually found congested, and the aggregate mucous follicles, denominated the glands of Peyer, in the ileum, are invariably ulcerated. Dr. Wood remarks, "that these ulcerations of the ileum are quite as characteristic of the disease in question, as the peculiar pustular eruption is of smallpox.'" "It has," says he, "in fact, come to be regarded almost as a necessary post-mortem test of the existence of the disease." These ulcerations generally commence in the lower part of the jejunum, and extend through the ileum, being most numerous in its lower part. The ulcerations in the jejunum are isolated; but those in the ileum are in patches, which sometimes run into each other, and spread over a considerable surface. They do not all mature at the same time, but are found in every stage of progress, from their incipiency, to a size that will measure an inch in diameter. We frequently find all the coats of the intestine involved, and sometimes entirely perforated by them.

There are a variety of opinions as to the cause that determines this ulceration of the glands of Peyer, and several theories have prevailed, all of which, however, are unsatisfactory to my mind. It is the opinion of some pathologists, that it is a poison circulating with the blood through the entire system, and which acts as a foreign stimulant, producing irritability of of the different tissues, but more especially of the glands of Peyer—inflammation is the consequence, and suppuration and ulceration follows. It is scarcely probable that this theory can be correct, although it presents some claims to plausibility, for a poison circulating with the blood through the entire system, would, it appears to me, be as apt to produce inflammation of any other tissue as of the gland of Peyer; besides, it has been proven by examining the bodies of persons who died of enteritis, that an ordinary inflammation of the ileum will not produce an ulceration of those glands. Other pathologists believe that Typhoid Fever is essentially inflammation of these glands, and all the other phenomena result from this affection just as gastric fever results from inflammation of the stomach; but this cannot be so, for if it was, the severity of the disease would be in proportion to the extent, depth, and number of the ulcerations, which experience has taught us is not the fact. Dr. Stokes, in a recent publication, says, that during an attack of Typhoid Fever a "Typhoid matter" is deposited in the mesenteric and Peyer's glands, that causes their ulceration, and that this Typhoid matter has no physical characters by which it may be distinguished from other products, but that its characters are vital. All the facts in this theory are assumed. It is unreasonable to suppose that this peculiar matter should be deposited in those glands, and no traces of it discoverable in any other part of the system; and it seems, also, to be drawing too much upon the imagination, to conclude that the matter, which he says has no physical characters to distinguish it from other products, has vital and specific properties. In the June number of the Nashville Medical and Surgical Journal, Dr. Madden has given a theory of ulceration of those follicles which has been adopted by many of my medical friends. He says—"the follicles of Lieberkühn which are scattered through the whole alimentary canal have ducts or open extremities, and the liver, pancreas and Brunner's glands open by ducts into the duodenum, but that Peyer's glands are without any ducts or orifices through which to discharge their contents. As this *shut* sack fills with its peculiar secretion the superimposed mucous membrane grows thinner, until finally, the sack is ruptured from over-distention, and likewise the portion of mucous membrane intervening between the sack and surface of the membrane." He says, further, "that having no duct, each, in order to get clear of their contents, creates a lesion of continuity of the walls of the sack and of the mucous membrane; and another peculiarity is, that nature, in healing this lesion obliterates the sack and a new set of sacks are formed. Thus we are led to conclude that each set of Peyer's glands serves only a temporary purpose, for as each fills its function it is destroyed, and others come into existence as their special secretion is required to be performed. This is the anatomy and manner of action, peculiar to each of the glands of Peyer, whether it exists in the solitary state or in the agminated form." He contends, that, as there are no ducts to discharge the contents of these glands, a lesion of the mucous membrane of the ileum is consequent upon their discharge, and notwithstanding the lesions are small, they require the effusion of lymph to heal them. But, owing to the vitiated state of the blood in Typhoid Fever, "a bad unorganizable kind of lymph is deposited, which, instead of healing the lesions facilitate the breaking down of the tissue, and will establish just such a condition as we usually denominate ulceration of the glands of Peyer." I agree with Dr. Madden, as to the anatomy and manner of action of the glands, but I do not believe that a vitiated state of the blood prevents the healing of the lesions, produced by the discharge of their contents. Besides, it is a disputed point with pathologists, as to whether the blood is vitiated or not. Dr. Wood says, "the blood drawn during life, often does not apparently differ from its condition in health, it coagulates firmly, and unless the disease is attended with some accessory inflammation, exhibits no buffy coat." If a vitiated state of the blood—admitting that it is vitiated-causes the ulceration of the glands of Peyer in enteric fever, why does it not likewise, produce it in typhus? We find it almost invariably in the former, but seldom, if ever, in the latter disease; and no one will deny that the blood is equally vitiated in typhus. So this ground is certainly untenable.

It is an established fact, that undue irritation of the trunks of nerves, as well as their division or destruction, will produce gangrene, ulceration, &c., in the parts to which the nerves are distributed. Now, is it not highly probable, that the exciting cause of Typhoid Fever, exerts a specific, morbid influence on the great sympathetic system of nerves; preventing sufficient nutrition in the parts to which they are distributed, and that the ulceration of the glands of Peyer; the enlargement and softening of the heart, liver, spleen, kidneys, and mesenteric glands are the consequences from that cause. For the disease is ushered in by a train of nervous symptoms, and evidences of deficient nervous action mark its progress, step by step, to death or convalescence. It is, in truth, a *nervous fever.* Dr. Wood declares, that "the name, nervous fever, is less inappropriate" than Typhoid Fever, or typhus mitior; and I am of the opinion it is the most appropriate name that can be given it. I have taken notes of over one hundred cases treated by myself, and observation has convinced me that, the sympathetic nervous system is *primarily* affected, and that all the results that follow are in consequence of that affection. The reasons why I am induced to think that the great sympathetic nerves are the ones first affected, are, because the parts usually found diseased in Typhoid Fever, are supplied with nerves principally from the gangleons of the great sympathetic. The ileum, in which are situated the glands of Peyer, the ulceration of which, is one of the first effects produced by the exciting cause of the fever, is supplied with nerves entirely from the great sympathetic. And the gangleons themselves are sometimes found softened and easily broken down in post-mortem examinations of such as die of the disease. The peculiar anatomy and physiology of Peyer's glands may facilitate their ulceration to some extent; for, being filled with their secretion, which they discharge, thereby producing lesions of the mucous membrane, which may require the effusion of lymph to heal them. But owing to the deficient nervous influence, the requisite force is not supplied and the lesions are not healed. The lesions so conditioned may serve as nuclei for the subsequent ulceration.

From what I have said you perceive that it is my opinion, that Typhoid Fever is synonymous with *nervous fever*, and that it is caused by a specific poison generated by chemical action in such substances as accumulate about human residences. This exciting cause, the nature of which I am unable to determine, exerts its specific influence primarily on the great sympathetic nerves, causing deficient nervous action in the parts supplied by them, consequently deficient nutrition, and consequent upon that condition, ulceration and destruction of the parts.

I come now, gentlemen, to the treatment of this protean form of disease. I shall give you the treatment that I have found most efficacious in alleviating it. The remedies in the first stage should generally be mildly antiphlogistic, as the symptoms are frequently of an active inflammatory character; but these measures should be cautiously used, as it is often the case, notwithstanding the apparently inflammatory nature of the symptoms, that they are deceptive; and that the powers of life sink rapidly under the mildest treatment of this kind. But, as a general rule, the patient will bear an aperient, and if the skin is very warm, the application of cold water, or vinegar and water in the form of a sponge bath, without danger; and frequently they are productive of much good. As there is always diarrhœa, or a strong tendency to it, the mildest aperient should be selected. I frequently give half grain doses of calomel, with three or four grains of dovers powders, once every three hours, until I have given four or five doses, and then move the bowels with castor oil. I have found this treatment answer very well in a large majority of cases. It is necessary that the bowels be thoroughly evacuated, as their contents coming in contact with their lining membrane, will often be productive of mischief. After the evacuation of the bowels, it is necessary to give laudanum or opium to quiet them; after which, the febrile symptoms should be combated with refrigerant diaphoretics. Some physicians advise general blood-letting, if the fever runs high, and the pulse is full and strong, but I cannot subscribe to the practice. I used it in my early practice in such case, but invariably with bad effects. I have long since abandoned it, and now rely on milder means. I have no objection, however, to moderate local blood-letting, and have often seen it productive of much benefit. Leeches or cups applied to the temples or back of the neck, if there is much headache, is often necessary. They may also be advantageously applied over the right iliac region. Wine of ipecac is the best diaphoretic; but you will often have to combine it with small doses of some of the preparations of opium to prevent its operating on the bowels.

I have sometimes in the commencement of this fever used the veratrum viride; but when I have used it in large enough doses to affect the pulse, it has always, in my hands, been followed by too much prostration. If there is any diarrhœa, give opium to stop it; or if there is a tendency to diarrhœa, give opium to correct it. I use opium in almost every stage of the disease. I have seen it relieve the severest headaches; and also relieve coma, or delirium, which appeared to be caused by determinations to the brain, but which were actually produced by insufficient cerebral excitement. It is certainly an indispensable remedy in this disease. It controls the bowels more effectually, and is much more soothing to their irritated surfaces than any of the astringents, (which I doubt the propriety of using under any circumstances, on account of their tendency to dry up the secretion,) and there is no danger to be apprehended from its narcotic effects. After the bowels are evacuated at the commencement of the fever, I disapprove of the use of any purgative—keep the bowels quiet, is my motto, and if it is desirable at any time to move them, use injections.

Commencing at this stage of the disease (the second week) there is generally an imperative call for support of the vital powers. Camphor, carb. ammonia, and opium is now a good prescription. I do not use enough to produce decided narcotism, but enough to keep the system moderately under their influence. The camphor and carb. ammonia answer both the purposes of stimulant and diaphoretic, while the opium guards the bowels, which should be watched like "the Roman vestals watched the sacred fire." If the propriety of using the above stimulants is doubtful, on account of the activity of the pulse, or other symptoms counter-indicating the use of stimulants, continue the wine of ipicac., but combine it with paregoric. I prepare a strong stimulating lineament, composed of tincture of capsicum, tincture of gum camphor, aqua ammonia and olive oil, which I have applied to the whole course of the spine, two or three times a day, and which I have found to produce an excellent effect. It relieves the stupor, delirium, and other nervous symptoms in a short time. If the bowels spring notwithstanding the efforts to prevent it, opium, with nitrate of silver are the best remedies. They should be continued until they stop the diarrhœa, unless the bowels continue running off so long that it would be unsafe to continue the remedy. The blistering stage is generally about the middle of the second week, frequently much earlier. Blisters should be applied to the abdomen over large surfaces, and if there is stupor or delirium, to the nape of the neck. They should be kept running until there is some evidence of the approach of convalescence. If the tongue now becomes very dry, and the bowels are tympanitic, commence the use of oil of turpentine. This is certainly one of the very best remedies in the latter stages of this disease. I have frequently seen the tongue become moist, the tympanitis banish as it were by magic, and other marks of decided improvement take place in a very short time under its use. The diet in Typhoid Fever should be of easy digestion and nourishing. Beef tea, or any other animal broths, provided they do not contain too much oleaginous matter are the best articles of diet. Every means to sustain the vital powers, particularly after the first week of the fever, should be used. Give stimulants, nourishing diet, and keep the bowels quiet and cases generally do well. There is no necessity to fear costiveness in this disease. I have seen patients rapidly improving, whose bowels had not acted in eight or ten days; and I have seen but few cases of Typhoid Fever die, when the physician was able to keep down diarrhœa. Susceptibility of the patient's bowels to be influenced by such remedies as are given to control them, is one of the most favorable symptoms met with in this disease. The above is the plan of treatment that I have found most efficacious in this Fever.

I have dealt in generals, in this part of my essay, deeming it unnecessary to enter into detail, as I had nothing new to advance. In fact, the general landmarks of treatment in this Fever are all that can be given, for it is impossible to give the treatment in every "collateral issue" that arises, unless we were acquainted with all the circumstances connected with the patient.

I have objections to the exclusive modes of treatment that have been recommended, which I will give and then I am done. It is the practice of some physicians to rely entirely on what is termed the "mercurial treatment." I object to this treatment, because, I have found it almost impossible to produce the specific effects of mercury in persons affected with this Fever. It will almost invariably cause the bowels to run off, and when it does not, it is followed during convalescence, which it does not hasten, by severe salivation and ulceration of the gums. And, besides those effects, it assists the disease to produce a vitiated state of the blood, to which many physicians attribute all the phenomena of Typhoid Fever.

The "antimonial practice" by Fordyce, I regard as decidedly dangerous and destructive. Nothing that I have used will produce as much irritation of the bowels in as short a length of time, as tartar emetic. Its use cannot possibly be persevered in, without producing uncontrollable diarrhœa that rapidly exhausts the patient. I have tried it under the most favorable circumstances, and always with the same sad results. As to the purgative plan of M. Delarrocque, it is certainly homicidal. I am certain, that if I were so to treat such cases as occur in this country, or such as it has fallen to my lot to witness, I would not cure one in a hundred patients; for, if there is any fact well established in the treatment of this disease, it is that purgatives are inadmissable. I have often seen it the case, that even a mild aperient administered after the first week of the Fever, when the bowels appeared to be constipated, was productive of fatal diarrhœa. What result then would follow the administration of purgatives in succession?

I will answer the question—death—certain death. All exclusive plans of treatment are objectionable, from the fact, that they are empirical, and take no account of the differences in the character of the disease in different persons, and the changes that it undergoes in all its successive stages.

I submit this thesis to your kind considerations—if there is anything in it that conflicts with your opinions and experiences, let your criticisms be mild, for He who hath made everything correctly, has not constituted men to think alike, or see alike.

